# Characterization of the composition, structure, and functional potential of bamboo rhizosphere archaeal communities along a chromium gradient

**DOI:** 10.3389/fmicb.2024.1372403

**Published:** 2024-04-17

**Authors:** Xiaoping Zhang, Qiaoling Li, Zheke Zhong, Zhiyuan Huang, Fangyuan Bian

**Affiliations:** ^1^Key Laboratory of State Forestry and Grassland Administration on Bamboo Forest Ecology and Resource Utilization, China National Bamboo Research Center, Hangzhou, China; ^2^National Long-term Observation and Research Station for Forest Ecosystem in Hangzhou-Jiaxing-Huzhou Plain, Hangzhou, China; ^3^Engineering Research Center of Biochar of Zhejiang Province, Hangzhou, China; ^4^Key Laboratory of High Efficient Processing of Bamboo of Zhejiang Province, Hangzhou, China

**Keywords:** chromium pollution, archaea, bamboo, rhizosphere, phytoremediation

## Abstract

**Introduction:**

Bamboo can be used in the phytoremediation of heavy metal pollution. However, the characteristics of the bamboo rhizosphere archaeal community in Cr-contaminated soil under field conditions remain unclear.

**Methods:**

In this study, high-throughput sequencing was used to examine the rhizosphere soil archaeal communities of Lei bamboo (*Phyllostachys precox*) plantations along a Cr pollution gradient.

**Results:**

The results revealed U-shaped relationships between Cr [total Cr (TCr) or HCl-extractable Cr (ACr)] and two alpha indices (Chao1 and Shannon) of archaea. We also established that high Cr concentrations were associated with a significant increase in the abundance of Thaumarchaeota and significant reductions in the abundances of Crenarchaeota and Euryarchaeota. The archaeal co-occurrence networks reduced in complexity with Cr pollution, decreasing the community’s resistance to environmental disturbance. *Candidatus nitrosotalea* and *Nitrososphaeraceae_unclassified* (two genera of Thaumarchaeota) were identified as keystone taxa. The community structure of soil archaeal communities was also found to be affected by TCr, ACr, pH, total organic C, and available nutrient (N, P, and K) concentrations, with pH being identified as the most reliable predictor of the archaeal community in assessed soils.

**Discussion:**

These findings enhance our understanding of microbial responses to Cr pollution and provide a basis for developing more refined approaches for the use of bamboo in the remediation of Cr-contaminated soils.

## 1 Introduction

Chromium (Cr) has drawn public attention owing to its toxic effects on soil, plants, and human beings (Sharma et al., 2021). The Cr in soil originates from both natural and human activities, such as fertilizer application, mineral exploitation, textile dyeing, and electroplating (Yang et al., 2019). Globally, more than 30,000 t of Cr has been released into the environment in the past 50 years, and most of it has accumulated in the soil, resulting in Cr pollution (Fallahzadeh et al., 2017). Li et al. (2020) indicated that the Cr content in China’s farmland soils is 1.48–820.24 mg/kg, and the contents at 4.31 and 0.12% of the sites they examined exceeded the screening (150 mg/kg) and control (800 mg/kg) values, respectively. In such cases, effective measures should be taken to mitigate and remediate the negative effects of Cr.

Bamboo forests cover the largest total forested area (1.0%) globally, with an area of 31.5 × 10^6^ ha (Li et al., 2018). Bamboo plants grow quickly, propagate rapidly, and can be cultivated easily (Cao et al., 2011). Bamboo also offers economic benefits owing to its high shoot yield (Li et al., 2010). Further, bamboo has positive ecological effects, such as carbon (C) cycling and climate mitigation (Cao et al., 2011; Terefe et al., 2019). Certain bamboo species can grow normally in heavy metal (HM) contaminated soil, although bamboo is not a hyperaccumulator, it is a strong candidate for use in HM phytoremediation (Bian et al., 2020).

Similar to fungi and bacteria, archaea not only play critical roles in biogeochemical cycles, including C, N, and S cycles (Yang et al., 2016), but they are also involved in HM transformation (Gilmour et al., 2018; Ni et al., 2020). Previous studies have shown that archaea are highly adaptable to HM stress and can convert toxic HM into less or non-toxic forms (Chaturvedi et al., 2015; Krzmarzick et al., 2018). Cr can reportedly affect soil archaeal community structures, abundances, and diversity (Li et al., 2017). The rhizosphere refers to the soil affected plant roots and the root tissue colonized by microorganisms (Hassan et al., 2019); these microbial communities are highly diverse, hosting genetic material more extensive than that of the host plant (Mendes et al., 2013). The community structure of these microbial communities influences nutrient cycling, plant growth, and root health in soil systems (Chepsergon and Moleleki, 2023). For example, these rhizosphere-linked microorganisms can promote the growth of plant roots by secreting iron carriers and plant hormones, improving nutrient utilization, and increasing the availability of HMs, thereby promoting plant absorption and accumulation of HMs (Zhu et al., 2023). In addition, rhizosphere microorganisms rapidly respond to environmental changes (Zhang et al., 2020; Sun et al., 2021), allowing them to efficiently collaborate with the host plant to maintain homeostasis (Bhanse et al., 2022). Soil–plant-microbiota is a closely interconnected biological network where various physiological and biochemical reactions occur between microbial communities, plant roots, and soil environment to promote energy flow, material cycling, and signal transmission in soil ecosystems, thus possessing great potential in plant remediation (Ma, 2019). Most studies delving into the phytoremediation of Cr-contaminated soils have only been conducted in laboratory settings (Xia et al., 2019); however, archaeal communities present in bamboo rhizosphere soil under field conditions have not been comprehensively examined.

In this study, archaeal community distributions were examined in the bamboo rhizosphere along a Cr gradient under field conditions. We hypothesized that Cr pollution can induce significant shifts in the soil archaeal communities of bamboo plantations, and consequently, these changes can affect other soil properties. The specific objectives of this study were to (1) characterize the archaeal communities and their functional profiles in bamboo rhizosphere soil exposed to different Cr concentrations under field conditions; and (2) determine the associations among soil parameters, Cr contents, and archaeal communities. We anticipate that the findings of this study will facilitate a greater understanding of the associations among Cr pollution, the soil chemical environment, and shifts in archaeal diversity and function. Moreover, these findings could contribute to the development of robust strategies for using bamboo in the phytoremediation of Cr-contaminated soils.

## 2 Materials and methods

### 2.1 Study site and sample collection

The soil was collected from the Lin’an District (30°18′ N, 119°34′ E) of Hangzhou, Zhejiang Province, China (Supplementary Figure S1). The study area was a Lei bamboo plantation (approximately 45 ha) that contained a 1–2 m wide stream. Owing to previous wastewater discharge (1995–2009) from a Cr-galvanizing plant, the bamboo plantation contains varying levels of HM pollution depending on the distance from the stream. The preliminary investigations were conducted to determine the status of soil Cr pollution in the studied area. After statistical analysis (one-way ANOVA with LSD *post-hoc* test), we selected five different levels of total Cr (TCr) concentrations for further analysis: low (L, 46.65 ± 0.68 mg/kg), low–moderate (LM, 77.64 ± 2.53 mg/kg), moderate (M, 87.23 ± 1.83 mg/kg), moderate–high (MH, 133.10 ± 1.56 mg/kg), and high (H, 357.38 ± 19.72 mg/kg). Five 3 × 3 m sampling plots were established for each treatment. Within each sampling plot, three bamboo plants were selected and dug out, and the rhizosphere soil of each bamboo plant was collected and mixed as a composite sample. Rhizosphere soils were collected using the method described by Zhou et al. (2013). Briefly, the roots of each bamboo plant were first gently shaken to remove the non-rhizosphere soil. The roots were then transferred to a sampling tube and shaken to collect soil tightly adhered to the roots, which represents the rhizosphere soil. A total of 25 fresh soil samples were taken, sieved with a 2 mm mesh to remove any stones, roots, and large organic residue, and then used in subsequent chemical and microbial analyses. The Cr concentrations in the L treatment were lower than the total average Cr in the agricultural area (48.0 mg/kg) (Yang et al., 2018), therefore, it was considered to be an uncontaminated soil.

### 2.2 Soil chemical analysis

The soil pH was determined with a glass electrode with a soil-to-water ratio of 1:2.5 (w:v). The soil total organic carbon (TOC) was analyzed using a TOC analyzer (Multi N/C 3100, Analytik Jena AG, Jena, Germany). Soil alkali-hydrolysable N (AN, alkali solution diffusion method), available phosphorus (AP, extracted with 0.5 M NaHCO3), available K (AK, extracted using 1 M ammonium acetate acid), and available Cr (ACr, extracted with 0.1 M HCl) were determined using methods described by Zhang et al. (2020).

### 2.3 DNA extraction and sequencing

DNA from the soil samples was extracted using the E.Z.N.A.^®^ Soil DNA Kit (Omega Bio-tek, Norcross, GA, United States) according to the manufacturer’s instructions. The archaeal diversity was estimated using archF (5′-TGYCAGCCGCCGCGGTAA-3′) and archR (5′-YCCGGCGTTGAVTCCAATT-3′) (Pires et al., 2012). For each of the 25 DNA samples, the polymerase chain reaction (PCR) amplification and sequencing were performed using a MiSeq PE300 platform (Illumina, San Diego, CA, United States) at LC-Bio Tech. Co., Ltd. (Hangzhou, China) with a minimum depth of 30,000 reads per sample. PCR amplification was conducted using a total reaction mixture volume of 25 μL. This mixture contained 25 ng of template DNA, 12.5 μL of PCR Premix, 2.5 μL of each primer, and sufficient PCR-grade solution buffer to adjust the volume. The PCR conditions comprised an initial denaturation at 98°C for 30 s, 32 cycles of denaturation at 98°C for 10 s, annealing at 54°C for 30 s, extension at 72°C for 45 s, and a final extension at 72°C for 10 min.

### 2.4 Sequence processing

The sequences were processed using the method described by Zhang et al. (2020). Briefly, FLASH (Magoč and Salzberg, 2011), Fqtrim,^1^ and the Vsearch tool (Rognes et al., 2016) were used for paired-end reads assembly, quality control, and chimera removal, respectively. DADA2 (Callahan et al., 2016) was used to generate the amplicon sequence variants (ASVs). In addition, taxonomy was assigned against the SILVA 132 database (Quast et al., 2013) using the q2-feature-classifier plugin in QIIME 2 (Bolyen et al., 2019). PICRUSt2 (Douglas et al., 2020) was used to predict the metabolic pathways of the archaeal communities.

### 2.5 Statistical analysis

One-way ANOVA and LSD *post hoc* test were conducted using IBM SPSS (version 19.0; Chicago IL, USA) to evaluate the differences in soil properties, alpha indices, and dominant taxa (with *p* ≤ 0.05 being significant). Alpha index calculations and clustering plot were performed in R using the “microeco” package (Liu et al., 2021). The “vegan” R package (Dixon, 2003) was used to evaluate the effects of the soil parameters on the archaeal communities via redundancy analysis (RDA). Principal coordinates analysis (PCoA) was performed using the “pcoa” function of the “ape” R package (Paradis et al., 2004). Analysis of similarities (ANOSIM) was conducted using the “vegan” R package (Dixon, 2003). The microbial networks were determined using the “WGCNA” (Langfelder and Horvath, 2008) and “igraph” (Csardi and Nepusz, 2006) R packages, and were visualized using Gephi (Bastian et al., 2009). Specifically, only ASVs present in 80% of all samples were used to construct the network. In addition, Spearman’s correlation coefficient > 0.6 and *p* ≤ 0.05 were used to characterize the dynamics of the resultant network. The keystone species were identified as the nodes with the highest betweenness centrality scores in co-occurrence networks (Vick-Majors et al., 2014). The R’s “ggcor” package (Huang et al., 2020) was applied to perform the Spearman’s correlation analysis and the mantel test. The Random Forest model was constructed to identify the key predictors of soil archaeal communities using the R package “randomForest” (Liaw and Wiener, 2002).

## 3 Results

### 3.1 Soil chemical environment as affected by Cr pollution

**Figure 1:**
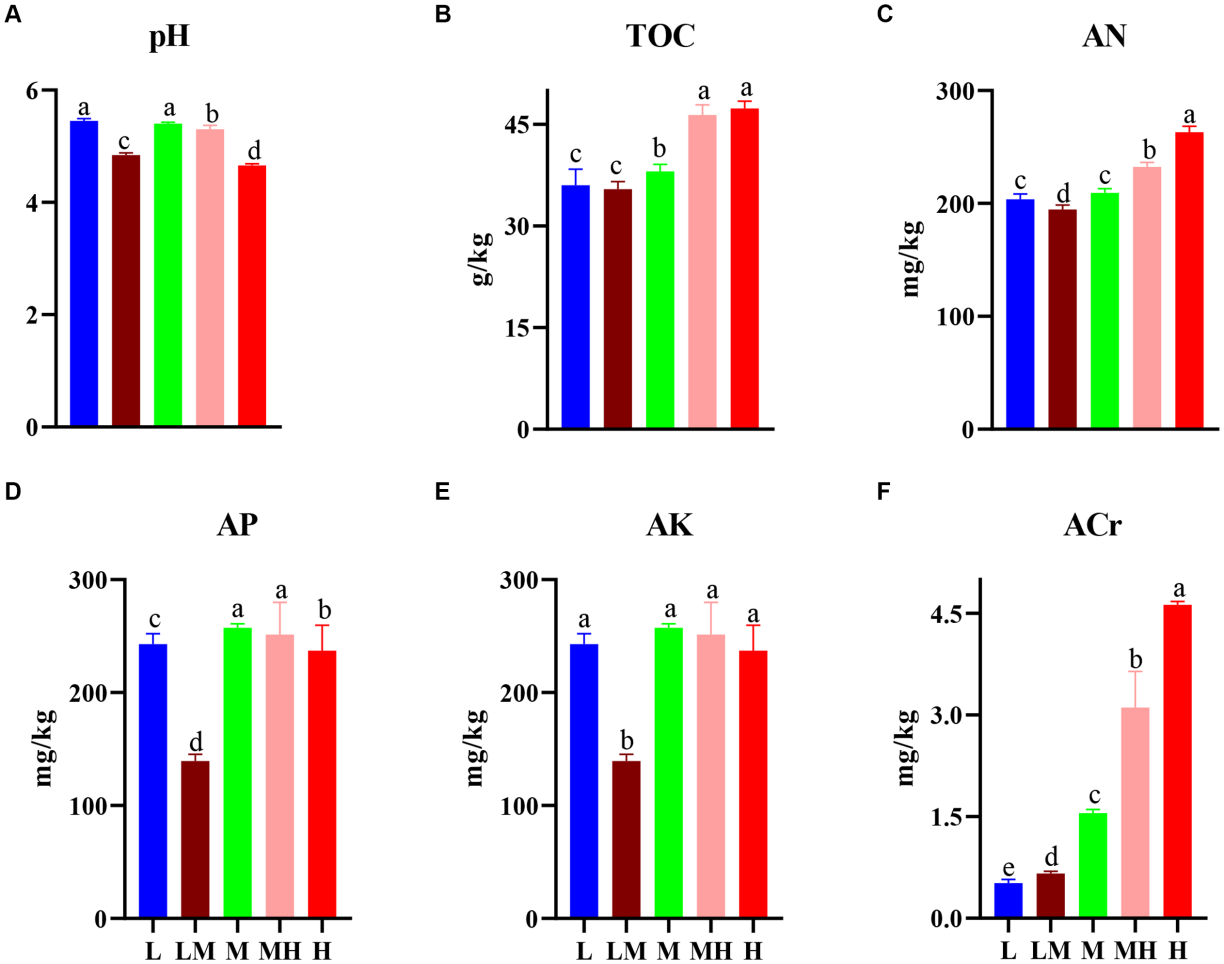
Changes in soil properties in the bamboo rhizosphere soils. (A-F) denotes the characteristics of soil pH, TOC, AN, AP, AK, and ACr in the bamboo rhizosphere respectively. The different letters indicate significant differences based on a one-way ANOVA (LSD, *p* ≤ 0.05). TOC, total organic carbon; AN, alkalihydrolysable nitrogen; AP, available P; AK, available K; L, low; LM, low-moderate; M, moderate; MH, moderate-high, H, high.

Compared with L, soil pH was significantly reduced in other four treatments (except M, Figure 1A), while TOC (except LM, Figure 1B), AP (except LM, Figure 1D), and ACr (Figure 1F) were significantly increased. H and MH treatments had the higher AN compared with other three treatments (Figure 1C). LM significantly decreased the AK relative to other four treatments (Figure 1E).

### 3.2 Archaeal diversity as affected by Cr pollution

**Figure 2:**
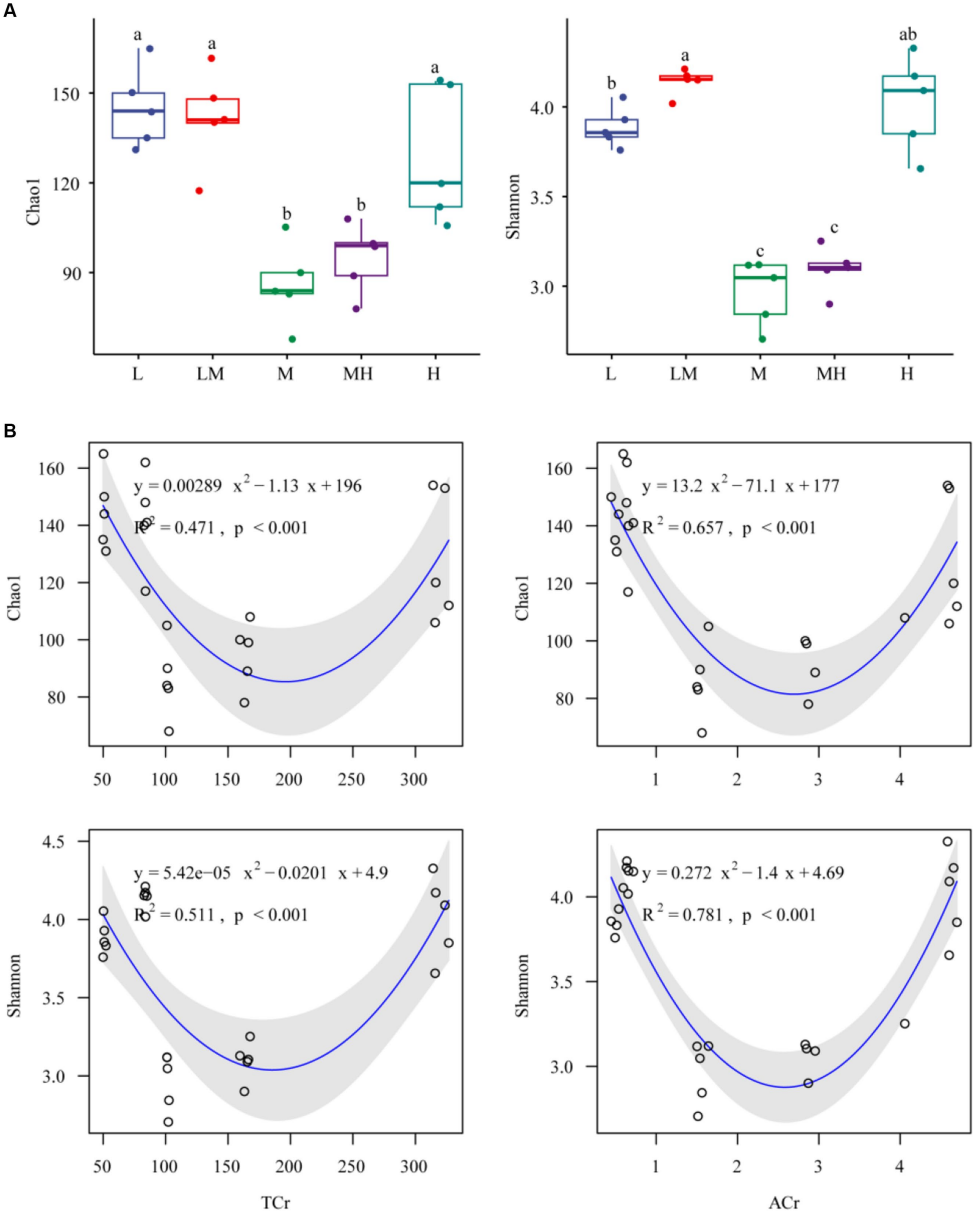
Alpha diversity indices of the soil archaeal microbiome exposed to different Cr concentrations **(A)**. Different letters indicate significant variation between the five Cr treatments (*p* < 0.05). L, low; LM, low-moderate; M, moderate; MH, moderate-high; H, high. Relationships between soil Cr and archaeal alpha indices **(B)**. TCr, total Cr; ACr, HCl-extractable Cr.

The archaeal Shannon and Chao1 indices in M and MH were significantly lower (*p* < 0.05) than those in the three other treatments (L, LM, and H) (Figure 2A). Both TCr and ACr showed significant non-linear trends with archaeal alpha diversity (both the Shannon and Chao1 indices, Figure 2B).

### 3.3 Archaea composition as affected by Cr pollution

**Figure 3:**
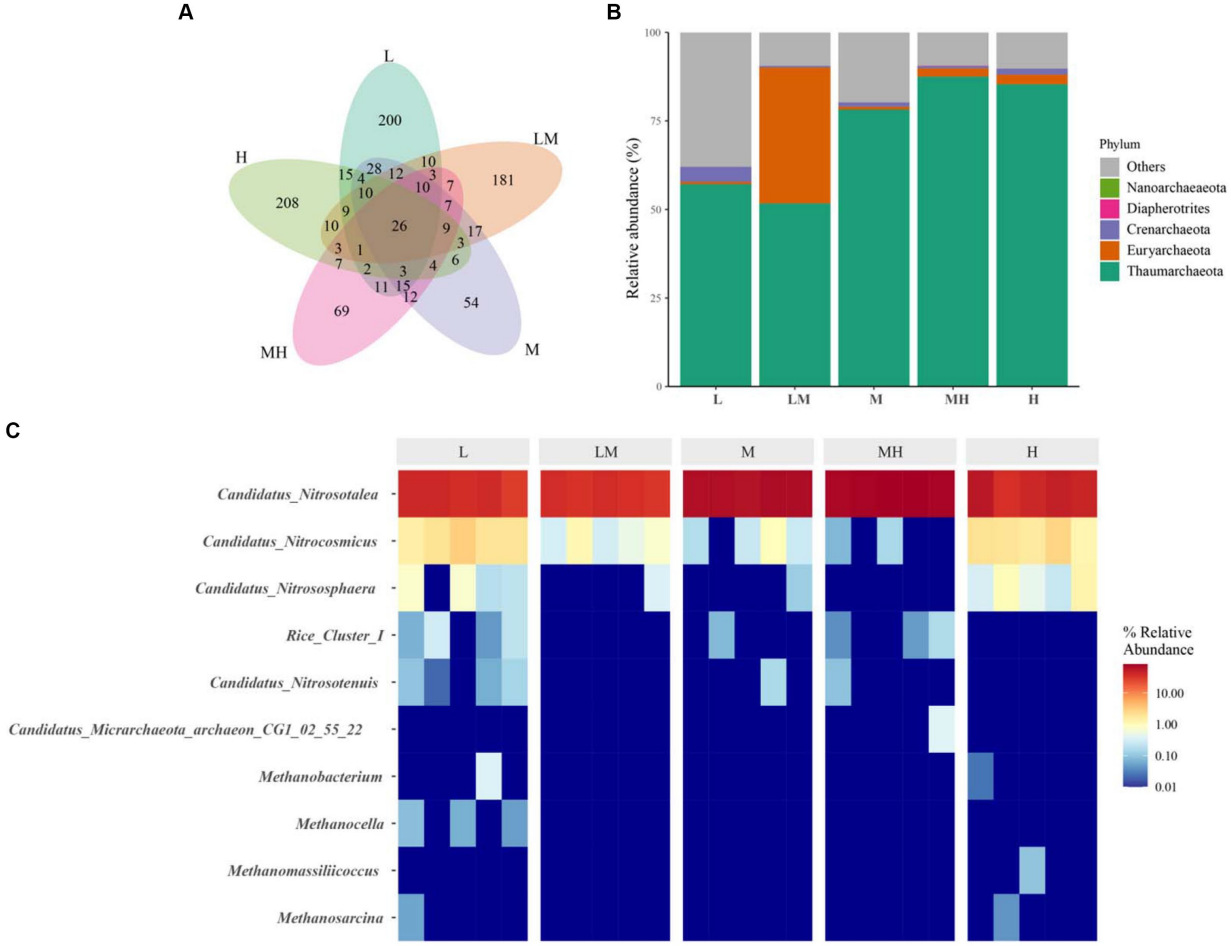
The profile of archaeal communities in Lei bamboo soils exposed to different Cr concentrations. **(A)** Venn diagram at the OTU level; **(B)** composition of archaeal communities at phylum level; **(C)** composition of archaeal communities at genus level. L, low; LM, low–moderate; M, moderate; MH, moderate–high; H, high.

**Figure 4:**
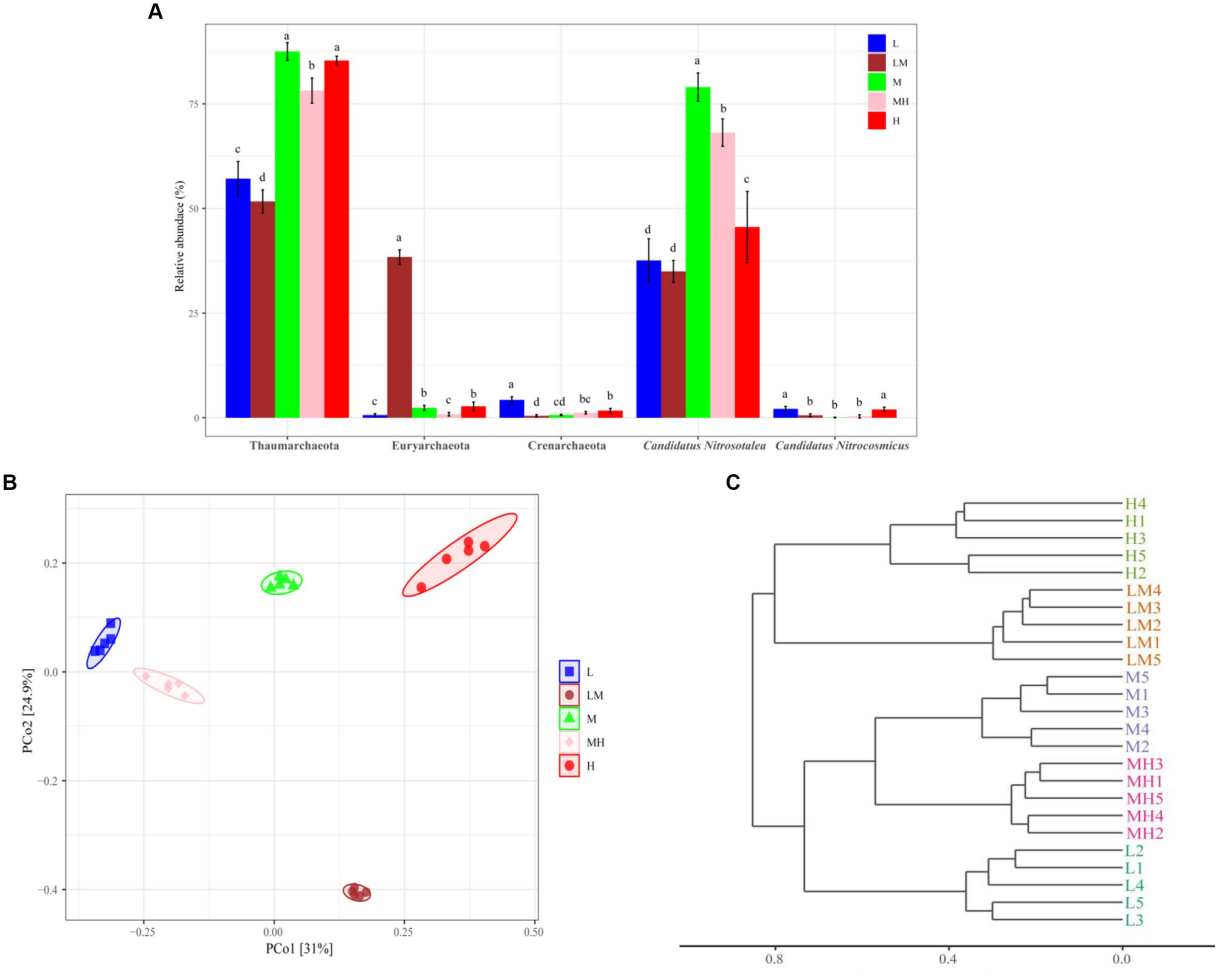
Comparative analysis of the compositions of dominant archaeal phyla and genera in Lei bamboo soil exposed to different Cr concentrations **(A)**. The different letters indicate significant differences based on a one-way ANOVA (LSD, *p* ≤ 0.05). PCoA of Bray–Curtis distances between all soil samples **(B)**. Hierarchical cluster analysis for soil archaeal communities **(C)**. L, low; LM, low-moderate; M, moderate; MH, moderate-high, H, high.

After quality control, a total of 459, 682 high-quality archaeal reads were obtained from all the soil samples. A total of 958 archaeal ASVs were identified. A total of 26 ASVs were shared among the five treatments, and 200, 181, 54, 69, and 208 ASVs were unique in the L, LM, M, MH, and H treatments, respectively (Figure 3A). Three archaeal phyla with more than 1% abundance were identified in the soil samples (Figure 3B), including Thaumarchaeota (71.95%), Euryarchaeota (8.99%), and Crenarchaeota (1.65%). Compared with L (Figure 4A), the relative abundance of Thaumarchaeota (except LM, H ≈ M > MH > L > LM) and Euryarchaeota (except MH, LM > H ≈ M > M ≈ L) in the other four treatments increased significantly (*p* < 0.05), while and the relative abundance of Crenarchaeota (L > H > M ≈ MH > LM) decreased significantly (*p* < 0.05).

At the genus level, two archaeal genera were dominant (> 1%) in the soils: *Candidatus nitrosotalea* (53.05%) and *Candidatus nitrocosmicus* (1.01%) (Figure 3C). As shown in Figure 4A, *Candidatus nitrosotalea* was significantly more abundant in the Cr-polluted treatments (except LM) than in L, in the order of M > MH > H > L ≈ LM, while *Candidatus nitrocosmicus* abundance was significantly lower in the Cr-polluted treatments (except H) than in L, in the order of L ≈ H > LM ≈ M ≈ MH (Figure 4A).

The PCoA (Figure 4B) revealed clear clustering of the archaeal communities from the five Cr pollution groups. PCoA1 and PCoA2 explained 31 and 24.91% of the variance, respectively (Figure 4B). The ANOSIM (*r* = 0.991, *p* = 0.001) and hierarchical clustering based on Bray–Curtis dissimilarity also indicated that the archaeal community differed significantly among the five treatments (Figure 4C).

### 3.4 Co-occurrence patterns of archaeal communities

**Figure 5:**
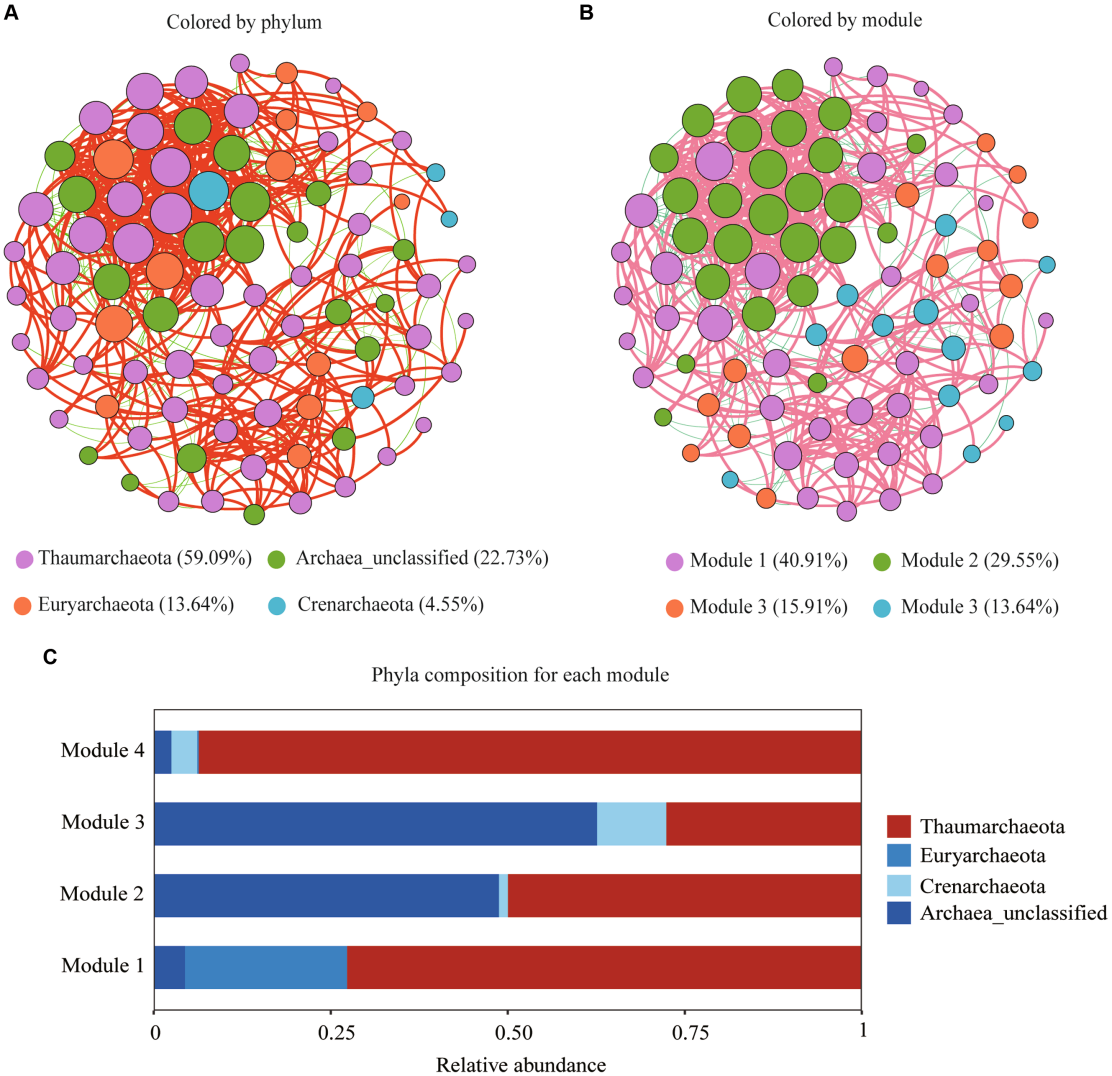
Archaeal community co-occurrence networks at the OTU level color-coded by phylum **(A)** and module **(B)**; Archaeal phyla composition for each module **(C)**.

The archaeal network was consisted of 88 nodes and 586 edges with a diameter of 6, average degree of 13.318, an average path length of 2.585, and a modularity of 0.969 (Figure 5A). The nodes of the network were assigned to three archaeal phyla (Thaumarchaeota, Crenarchaeota, and Euryarchaeota), accounting for 77.27% of all the nodes, with 22.73% of them remaining unidentified at the phylum level (Figure 5A). The top three ASVs were identified as keystone taxa and assigned to two Thaumarchaeota genera: *Candidatus nitrosotalea* and Nitrososphaeraceae_unclassified. Based on the modularity index, all nodes were classified into four modules (Figure 5B). Thaumarchaeota was the most dominant phylum in modules 1, 2, and 4, while archaea_unclassified dominated module 3 (Figure 5C).

**Figure 6:**
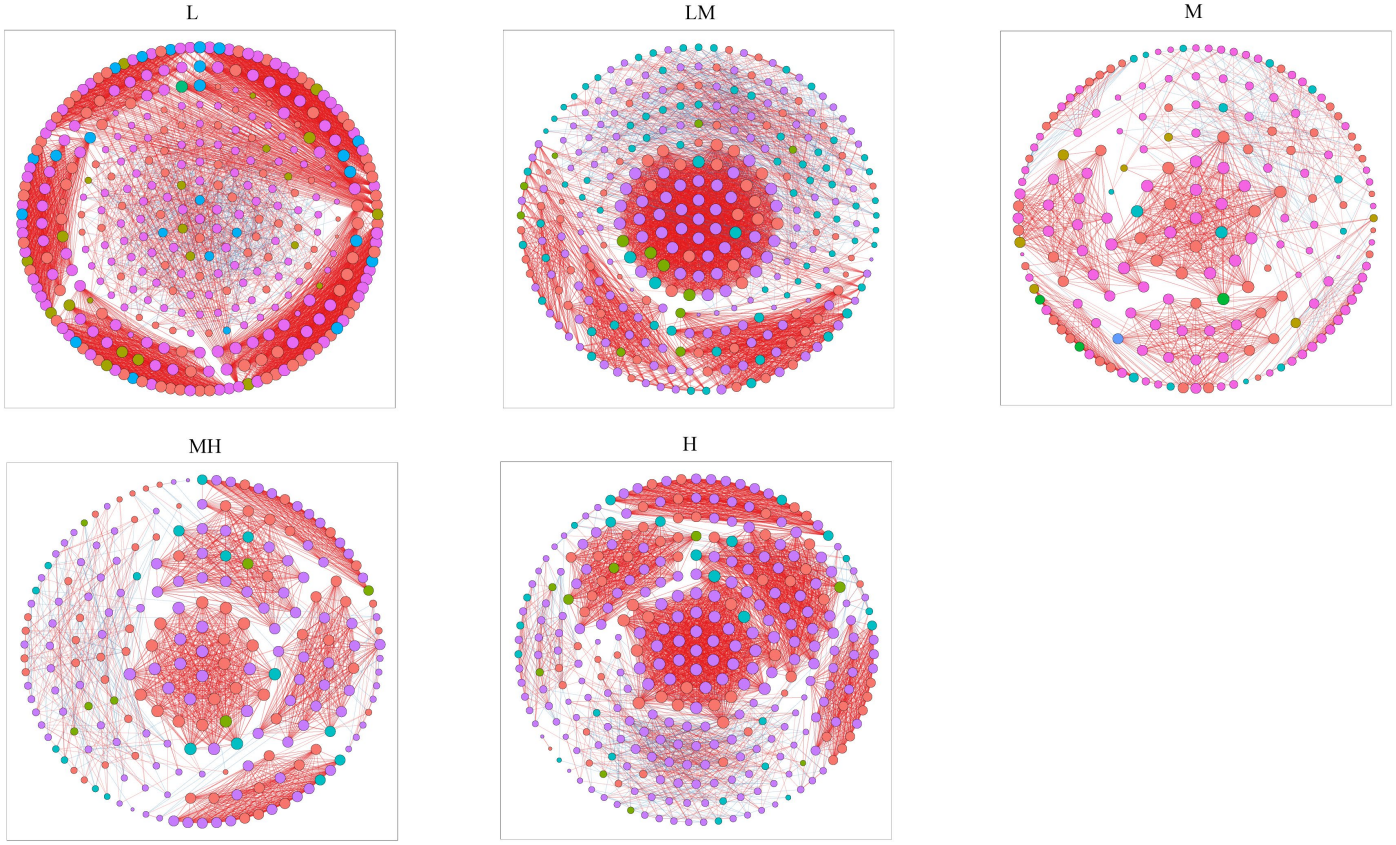
Co-occurring network of archaeal communities at the ASV level along a Cr gradient. The nodes are colored by phyla, and the connections denote a strong (Pearson’s |ρ| > 0.6) and significant (*p* < 0.05) correlation.

Co-occurrence networks were also determined for archaeal communities at each Cr level based on the top 500 ASVs (Figure 6). The co-occurrence network analysis showed that the number of nodes and edges in the L network were higher than in the other four (LM, M, MH, and H) networks (Figure 6; Supplementary Table S1), indicating that Cr pollution reduced the network complex. The number of positive edges in the L, LM, M, MH, and H networks were 4,495, 3,910, 1,094, 1,704, and 3,786, respectively, accounting for 92.76, 90.95, 91.01, 93.32, and 92.48% of the total number of edges (Figure 6; Supplementary Table S1).

### 3.5 Predicted functional potential of the soil archaeal community

**Figure 7:**
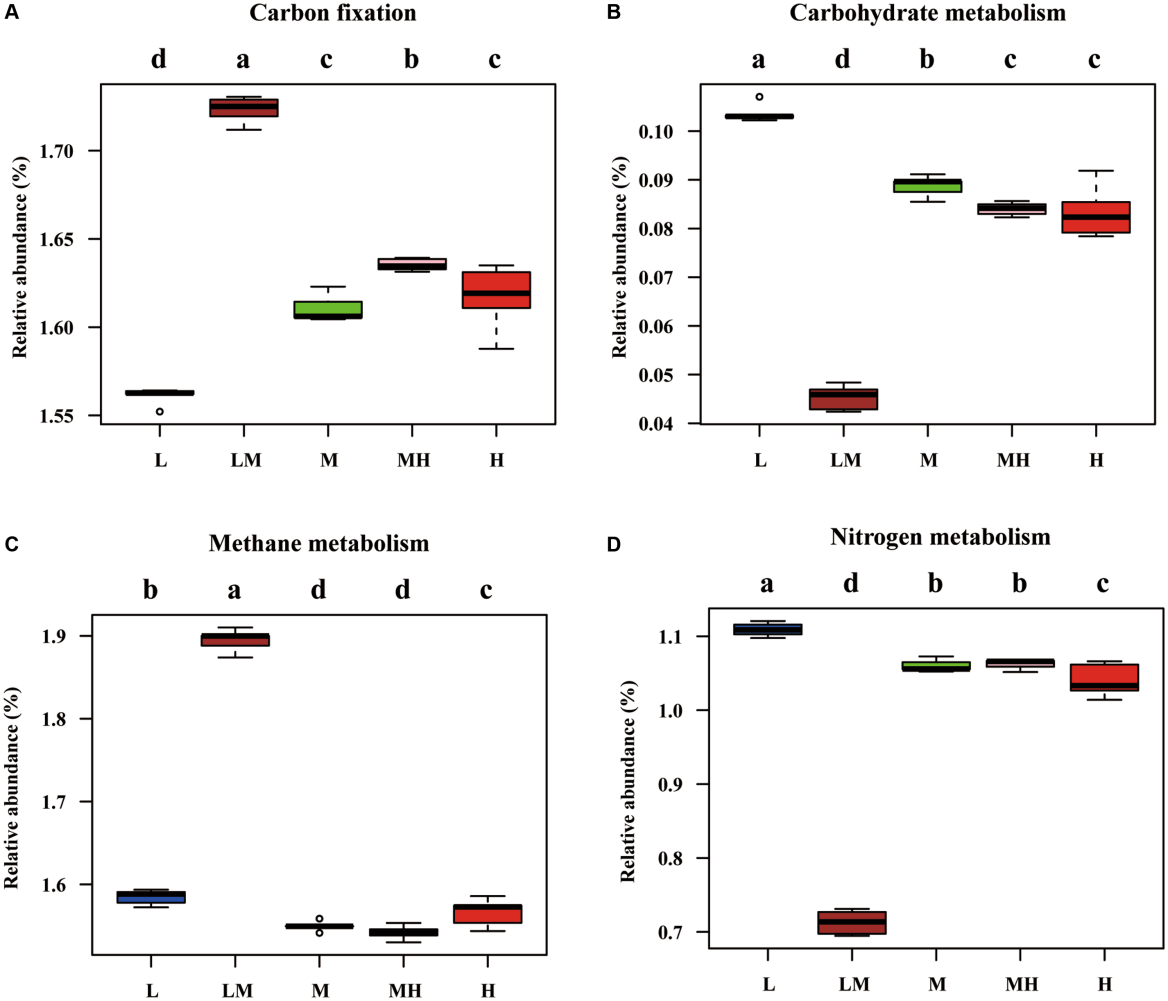
Pathways related to nitrogen and methane metabolism, as predicted by the Kyoto Encyclopedia of Genes and Genomes (KEGG). (A) Carbon fixation; (B) Carbohydrate metabolism; (C) Methane metabolism; (D) Nitrogen metabolism. The different letters indicate significant differences among the five Cr levels based on a one-way ANOVA (LSD, *p* ≤ 0.05).

The PICRUSt2 analysis indicated that there were 28 functional genes with a relative abundance of more than 1% (Supplementary Figure S1), including general function prediction only (5.35%), ribosome (5.02%), urine metabolism (3.53%), pyrimidine metabolism (2.87%), amino acid-related enzymes (2.54%), transporters (2.23%), aminoacyl-tRNA biosynthesis (2.23%), DNA repair and recombination proteins (2.16%), and oxidative phosphorylation (2.16%). The pathways related to the C and N cycles were further analyzed, as shown in Figure 7. Compared with L, the abundance of the metabolic pathways for fatty acid metabolism (except LM), glycolysis/gluconeogenesis (except MH), and C fixation were significantly increased in the other treatments, while the abundance of the metabolic pathways for carbohydrate metabolism, methane metabolism (except LM), and N metabolism were significantly decreased.

### 3.6 Factors shaping the archaeal community

**Figure 8:**
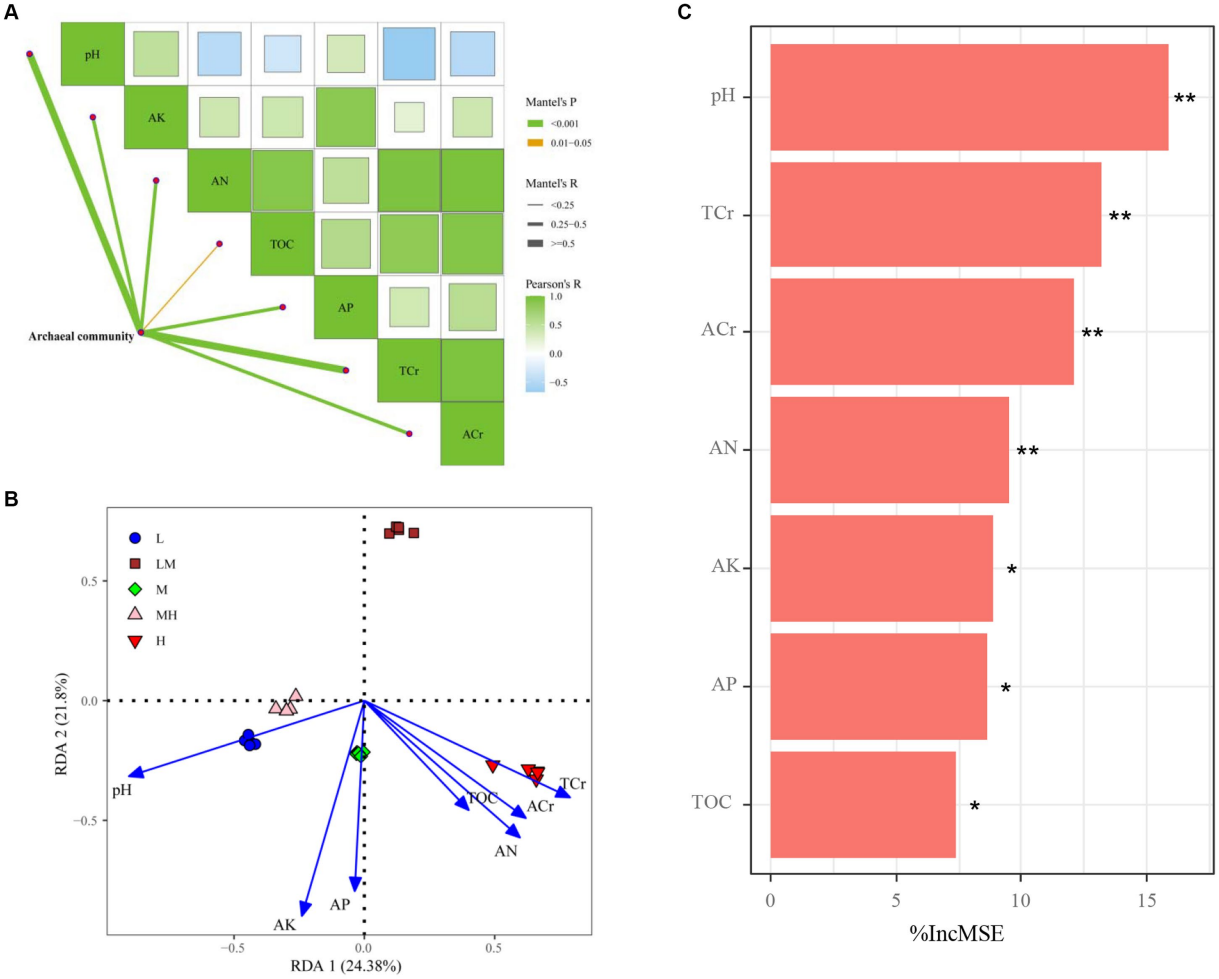
Spearman’s correlation analysis and Mantel tests for microbial communities **(A)**; edge width corresponds to the Mantel’s R value and the edge color denotes the statistical significance. Redundancy analysis of soil properties and archaeal communities **(B)**; random forest model identified the impact factors of microbial nutrient limitation in the studied soils **(C)**. **p ≤ 0.01; *p ≤ 0.05; IncMSE, the increase in mean square error. TOC, total organic carbon; AN, alkalihydrolysable nitrogen; AP, available P; AK, available K; L, low; LM, low-moderate; M, moderate; MH, moderate-high, H, high.

The Mantel test suggested that pH, TOC, AN, AP, AK, TCr, and ACr had important effects on the variation observed in the archaeal communities (Figure 8A). The RDA results indicated the first two axes explained 45.18% of the variability between the soil properties and the archaeal community (Figure 8B). The soil pH (*r*^2^ = 0.881, *p* = 0.001), TOC (*r*^2^ = 0.359, *p* = 0.009), AN (*r*^2^ = 0.662, *p* = 0.001), AP (*r*^2^ = 0.625, *p* = 0.002), AK (*r*^2^ = 0.852, *p* = 0.001), TCr (*r*^2^ = 0.756, *p* = 0.001), and ACr (*r*^2^ = 0.603, *p* = 0.001) were significantly associated with the archaeal community. Furthermore, pH exhibited the highest contribution to archaeal community revealed by random forest model (Figure 8C).

## 4 Discussion

### 4.1 Effects of Cr pollution on soil chemical environment in the bamboo rhizosphere

The results showed high Cr pollution had a decreasing pH as well as an increasing TOC compared with the uncontaminated area (Figures 1A,B). Studies indicated that pH is the most dominant factor driving HM mobility and bioavailability (Araújo et al., 2019; Liu et al., 2022). Wang et al. (2006) showed soil pH had a negative correlation with metal availability. Soil TOC was also found to increase the HM solubility and mobility as it provides organic chemicals and acts as chelating agents (Shrestha et al., 2007). Therefore, the decreased pH and increased TOC may contribute to the increased HCl-extractable Cr with the Cr accumulation. In addition, high Cr-polluted soils were found to have higher AN and AP than in the uncontaminated soil. Bian et al. (2018) suggested soil nutrients, such AN and AP, which were positively correlated with HM contents, indicating HMs can be removed by the uptake of plants.

### 4.2 Effects of Cr pollution on soil archaeal community composition in the bamboo rhizosphere

This study used deep 16S amplicon sequencing to investigate archaeal community characteristics in bamboo plantation soils at different Cr pollution levels. Our results indicate that increasing the Cr content altered the alpha diversity of archaeal communities in the bamboo rhizosphere, following a non-linear correlation. The archaeal alpha diversity presented a U-shaped distribution (first decreasing and then increasing) with increasing Cr content. In contrast, Cr contents negatively linear correlation with the bacterial alpha indices in the bamboo rhizosphere (Zhang et al., 2020). These phenomena may be caused by archaea perceiving and using environmental resources in more restrictive ways than bacteria do (Aller and Kemp, 2008). Furthermore, we found that the detected increase in archaeal alpha diversity in soil with a higher Cr content was associated with soil physicochemical properties (Jiao et al., 2020; Qiu et al., 2021). Soils with a higher Cr content were found to have higher levels of TOC, AN, and AP, which not only provided nutrients for the growth and proliferation of archaeal communities but also contributed to reductions in the mobility, toxicity, and bioavailability of HM (Peng et al., 2009; Bolan et al., 2014).

We found that Cr accumulation increased Thaumarchaeota abundance. Members of the phylum Thaumarchaeota are involved in N and C cycling (Walker et al., 2010). The increased abundance of this phylum in the Cr-polluted soil may be associated with increased TOC and AN. The co-occurrence network analysis also indicated that Thaumarchaeota was the most abundant phylum, and two Thaumarchaeota genera (*Candidatus nitrosotalea* and Nitrososphaeraceae_unclassified) were identified as the top three keystone taxa based on their BC scores. González et al. (2010) indicated that nodes with high BC scores play key roles in maintaining the connectivity of ecological networks. Therefore, in the bamboo rhizosphere, Thaumarchaeota might play a critical role in maintaining archaeal community structure and function under Cr pollution.

The results suggest that high Cr pollution decreased the abundance of Euryarchaeota, which is consistent with the findings of Li et al. (2017). Miranda et al. (2019) found that Euryarchaeota were negatively correlated with Cr, pH, TOC, K, and P. Therefore, the increase in the TCr, as well as the shifts in the soil chemical parameters, contributed to the decrease in Euryarchaeota abundance. In our study, the Crenarchaeota abundance was also decreased with high Cr pollution. Many studies have indicated that Crenarchaeota play important roles in ammonia oxidation (Nicol and Schleper, 2006) and autotrophic CO_2_ fixation (Shi et al., 2023). This indicates that the decrease in the Crenarchaeota can be partly explained by shifts in TOC and AN.

We also found that *Candidatus nitrosotalea* and *Candidatus nitrocosmicus* were dominant in the bamboo soils under Cr pollution. These two species are related to nitrification and ammonia oxidation (Herbold et al., 2017; Gornish et al., 2020), suggesting the two genera might play vital roles in N cycling under Cr pollution. In this study, with increasing Cr levels, the abundance of *Candidatus nitrosotalea* (except in LM) increased significantly, while that of *Candidatus nitrocosmicus* (except in H) decreased significantly. These findings suggest that high Cr pollution may affect N metabolism in the bamboo soils.

The results also indicated that Cr pollution decreased the complexity of the archaeal network, partly because high HM concentrations can affect the structure and function of cells and inhibit microbial activity or competitiveness (Shuaib et al., 2021). de Vries et al. (2018) suggested that more complexity of microbial networks will enhance the stability of the community and increase the ability of microbial communities to resist environmental disturbances. The changes in the complexity of archaeal networks indicate that Cr pollution can affect archaeal abundance and diversity and reduce community stability.

### 4.3 Effects of Cr pollution on the potential functions of the archaeal community

To date, the functional metabolic potential of archaeal communities in bamboo soils under HM contamination has not been well documented. In the current study, based on the PICRUST2 results, we found that high Cr levels were associated with higher levels of fatty acid metabolism, glycolysis/gluconeogenesis, and C fixation metabolic pathways and lower levels of carbohydrate metabolism, methane metabolism, and N metabolism pathways. According to Zhang et al. (2020), high Cr contamination levels (M, MH, and H) were associated with higher soil TOC and AN contents. In addition, archaea participate in C and N cycling (Offre et al., 2013). Nahar et al. (2020) reported that the net nitrification rate was significantly (*p* < 0.05) positively correlated with ammonia-oxidizing archaea and HMs (Fe, Pb, and Zn), however it was significantly (*p* < 0.05) negatively correlated with Cu. Thus, changes in the metabolic pathways related to C and N cycling in the archaeal community may contribute to soil TOC and AN accumulation.

### 4.4 Factors shaping the archaeal community and implication for phytoremediation

Although several HM ions play a vital roles in microbial metabolism at low concentrations (Appenroth, 2010), HM pollution is deleterious to the overall functioning of the biomass (Tang et al., 2022; Sun et al., 2023). Moreover, microbial communities have a strong selective pressure in response to HMs (Laplante et al., 2013). The RDA and Mantel test indicated that changes in soil TCr, ACr, pH, TOC, and available nutrients (AN, AP, and AK) caused changes in the archaeal communities. Furthermore, the random forest analyses suggested that pH was the primary predictor of archaeal community in the bamboo plantations with Cr pollution. This result supported the views from Gubry-Rangin et al. (2010), who revealed that pH plays a dominant role in driving the abundance and distribution of soil archaea. A slight increase in soil pH can cause a decrease in soil Cr mobility and reduce its bioavailability to plants (Shahid et al., 2017; Lin et al., 2022), which may be helpful in reducing Cr stress on soil microbes. These results may be partly due to relatively narrow pH ranges for optimal microbial growth (Rousk et al., 2010). Moreover, soil pH indirectly influences microbial communities by affecting substrate availability (Kemmitt et al., 2006), and imposes strong selective pressures that shape the total archaeal and ammonia-oxidizing archaeal communities (Tripathi et al., 2015). Based on the above results, it is feasible to increase the efficiency of bamboo phytoremediation by regulating the archaeal community via controlling the soil pH.

### 4.5 Limitations and future prospects

Despite the important findings of this study, it does have certain limitations. Notably, there are limitations with respect to the methods we adopted for microbial functional analysis. For example, whereas PICRUST2-based analysis can be used to predict the presence of pathways/genes, it is unable to predict actual functional performance (Douglas et al., 2020). In contrast, metagenomics does not require the isolation and cultivation of environmental microorganisms, and can facilitate the direct analysis of environmental microbial DNA to obtain information on the genetic, functional, and ecological characteristics of microbial communities (Huang et al., 2022). Moreover, metatranscriptomics can be applied to examine the types and quantities of all RNA transcribed by microbial communities in the contexts of specific environments, periods, and conditions, thereby enabling determinations of the metabolic functions of active microorganisms (Sharuddin et al., 2022). Accordingly, in further studies, we would hope to adopt a more omics-based approach to characterize the diversity and functional changes in archaeal communities.

## 5 Conclusion

Our findings in the study revealed significant changes in the archaeal communities of Cr-contaminated soil, including an increase in the relative abundance of Thaumarchaeota, and reductions in the abundances of Crenarchaeota and Euryarchaeota in soils with elevated concentrations of Cr. Moreover, we established that the composition of the archaeal community was significantly associated with soil TCr, ACr, pH, TOC, and available nutrient (AN, AP, and AK) contents, with soil pH being found to be the most reliable predictor of archaeal community structure. Furthermore, Cr pollution was found to alter soil archaeal functions associated with C and N cycling processes. These findings provide important insights into the overall distribution of archaea along a chromium gradient, and thereby make a valuable contribution to our current understanding of the ecology of archaeal communities inhabiting heavy metal-contaminated environments. These findings can provide a basis for designing appropriate strategies for applying bamboo in the phytoremediation of Cr-contaminated soils.

## Data availability statement

The datasets presented in this study can be found in online repositories. The sequencing data have been deposited in NCBI under Bioproject PRJNA991260.

## Author contributions

XZ: Writing – original draft, Investigation, Funding acquisition, Conceptualization. QL: Writing – original draft, Investigation. ZZ: Writing – review & editing, Funding acquisition, Conceptualization. ZH: Writing – original draft, Investigation. FB: Writing – original draft, Investigation, Funding acquisition.

## Footnotes

^1^ https://ccb.jhu.edu/software/fqtrim/
