# Supplementary material for: Characterization of the composition, structure, and functional potential of bamboo rhizosphere archaeal communities along a chromium gradient
Source: Front Microbiol. 2024 Apr 17;15:1372403. doi: 10.3389/fmicb.2024.1372403 (PMC11061513; doi:10.3389/fmicb.2024.1372403)
Supplement: Supplementary file 1 [file Data_Sheet_1.pdf]

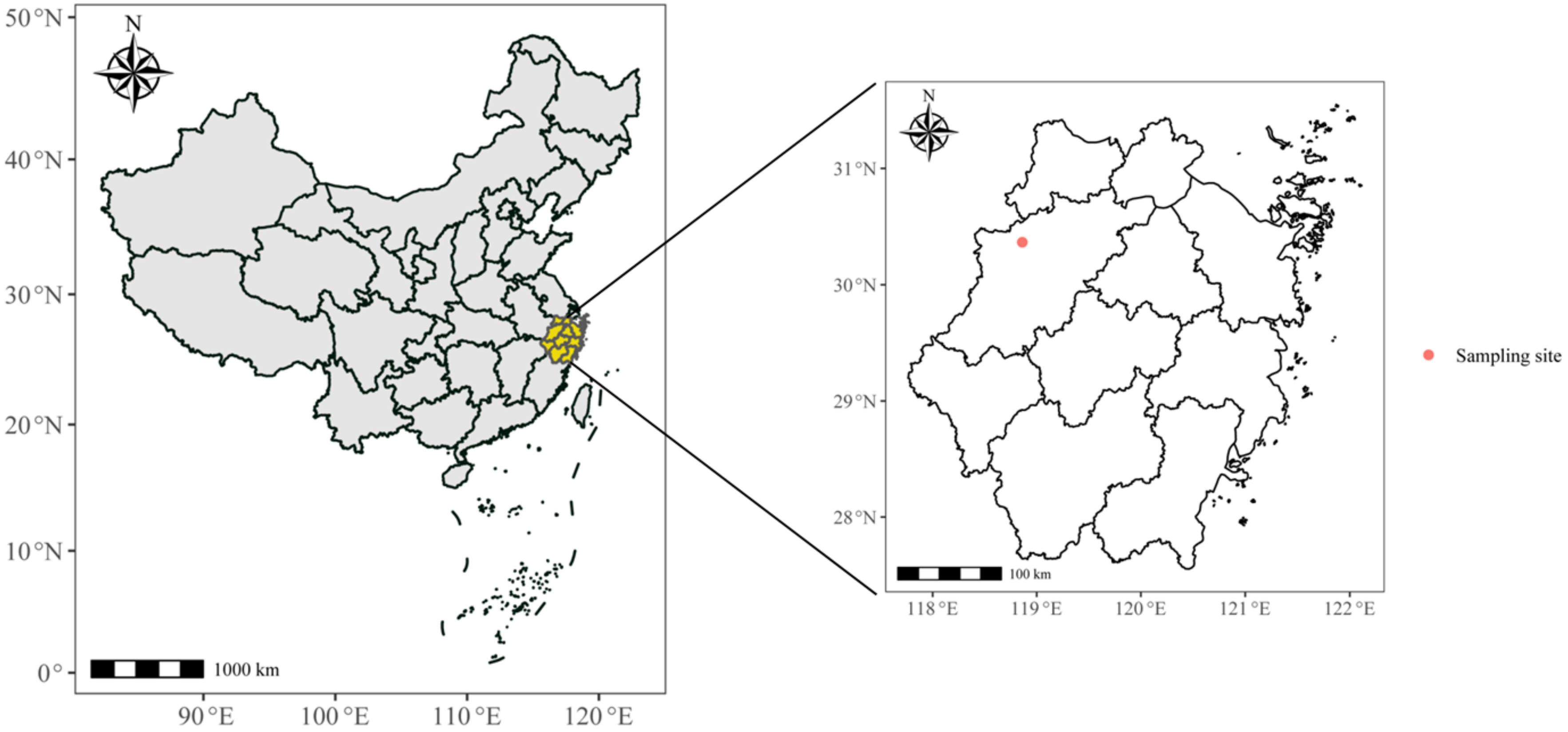

Figure S1 Location of the sampling site.

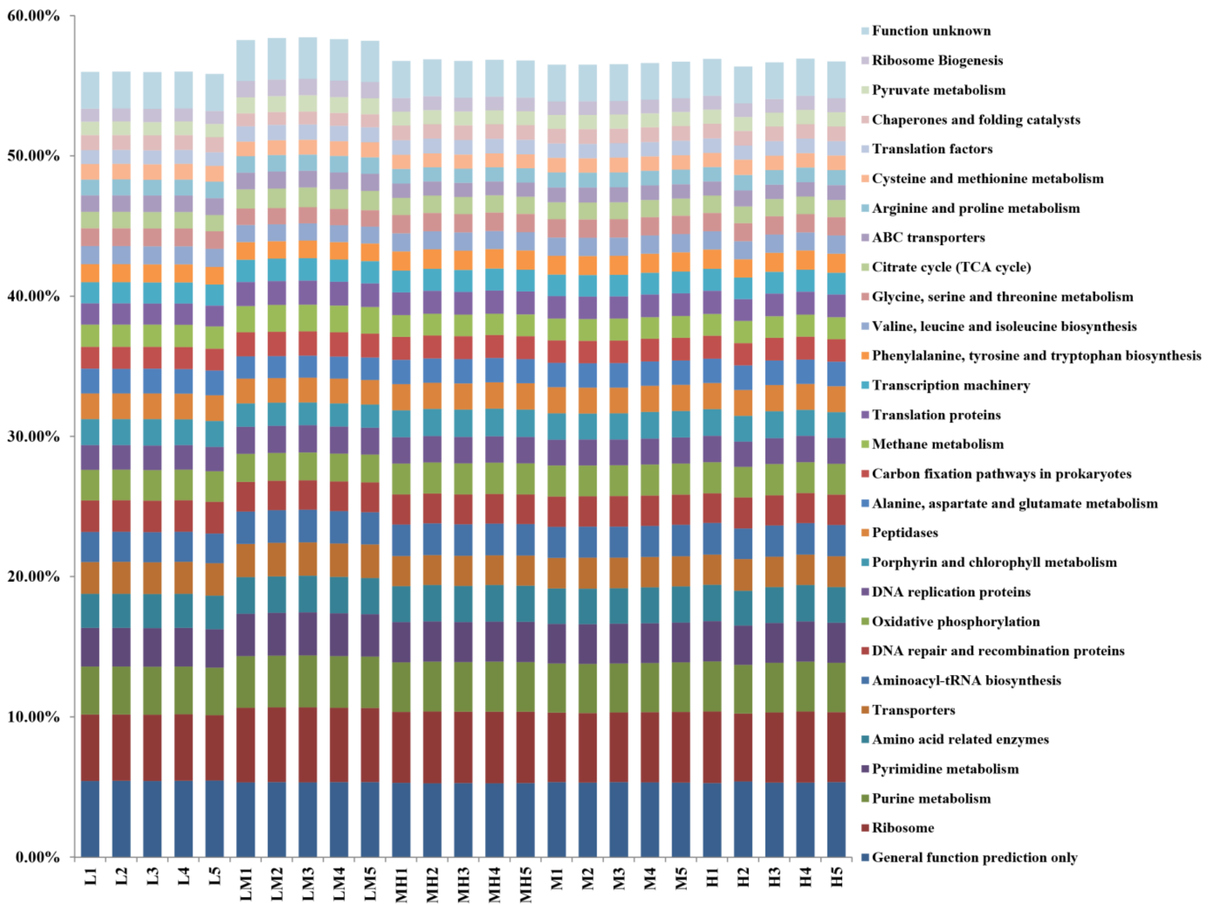

Figure S2 Predictive abundances of functional genes of archaeal communities obtained from PICRUSt results. Only functional genes with more than <1% abundance are shown.

Table S1 Archaeal network properties in the different Cr levels

	L	LM	M	MH	H
Node	359	318	189	220	320
Edge	4846	4299	1202	1826	4094
Average degree	27.00	27.04	12.72	16.60	25.59
Average path length	2.59	2.60	3.34	3.25	2.72
Clustering coefficient	0.97	0.98	0.94	0.98	0.98
Positive correlation	92.76%	90.95%	91.01%	93.32%	92.48%
Negative correlation	7.24%	9.05%	8.99%	6.68%	7.52%
